# Recent reduction in the water level of Lake Victoria has created more habitats for *Anopheles funestus*

**DOI:** 10.1186/1475-2875-7-119

**Published:** 2008-07-03

**Authors:** Noboru Minakawa, Gorge Sonye, Gabriel O Dida, Kyoko Futami, Satoshi Kaneko

**Affiliations:** 1 Institute of Tropical Medicine, Nagasaki University, Nagasaki, Japan; 2 International Centre for Insect Physiology and Ecology, Mbita, Kenya; 3 School of Public Health, Maseno University, Maseno, Kenya

## Abstract

**Background:**

The water level of Lake Victoria has fallen more than 1.5 m since 1998, revealing a narrow strip of land along the shore. This study determined whether the recent drop in the water level has created additional breeding grounds for malaria vectors.

**Methods:**

The recent and past shorelines were estimated using landmarks and a satellite image. The locations of breeding habitats were recorded using a GPS unit during the high and low lake water periods. GIS was used to determine whether the breeding habitats were located on newly emerged land between the new and old shorelines.

**Results:**

Over half of the breeding habitats existed on newly emerged land. Fewer habitats for the *Anopheles gambiae *complex were found during the low water level period compared to the high water period. However, more habitats for *Anopheles funestus *were found during the high water level period, and they were all located on the newly emerged land.

**Conclusion:**

The recent reduction in water level of Lake Victoria has increased the amount of available habitat for *A. funestus*. The results suggest that the water drop has substantially affected the population of this malaria vector in the Lake Victoria basin, particularly because the lake has a long shoreline that may harbour many new breeding habitats.

## Background

**Figure 1:**
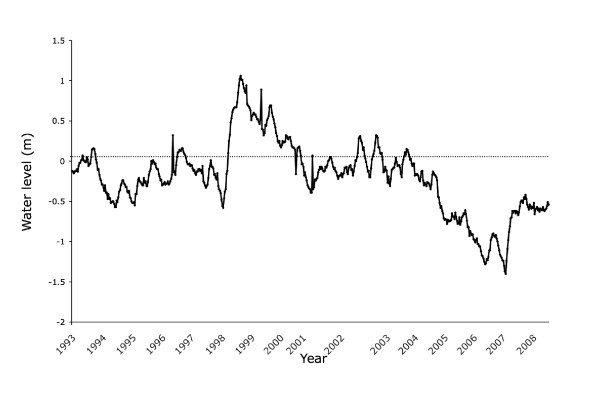
**Water level of Lake Victoria relative to the 10-year average from September 1992**. More data points were available in 2002; thus, the interval is wider than for other years. Radar altimeter data were obtained from the NASA/CNES Topex/Poseidon and Jason-1 satellite missions.

Satellite radar altimeter data [1] indicate that the water level of Lake Victoria in May 1998 was 1.06 m above its 10-year average from 1992 to 2001 (Figure 1). The rise was apparently due to the high amount of rainfall in 1997 and early 1998 [2,3], which was likely generated by the El Niño Southern Oscillation (ENSO) and the Indian Ocean Dipole (IOD) [4,5]. Subsequently, the water level began to drop and reached 1.4 m below the average in October 2006. Although the water level rose considerably after heavy rainfall related to ENSO and IOD in late 2006, it remained near 0.5 m below the average (1.5 m below the water level in 1998) in early 2008. The reduction in water level can be attributed in part to drought and the increasing outflow at the expanded Owen Falls dam in Uganda [6,7].

As the lake water level has declined, a narrow strip of land has appeared along the shore. This newly emerged land space may become a potential breeding ground for malaria vectors. Seepage water from nearby hills and the lake often create numerous stagnant water pools on the lakeshore, and waves sometimes feed the pools with lake water [8]. This study investigated whether the recent reduction in the water level of Lake Victoria has created more breeding habitats for malaria vectors.

## Methods

### Study area

The study area was approximately 3.8 km^2 ^and was surrounded by lake water to the north, west, and east. The elevation increases gradually toward a hill in the southern area of the site. The hill is approximately 1,300 m above sea level, and the lake surface was approximately 1,134 m above sea level in early 2008. There were no permanent streams and reservoirs, and the population of the area was approximately 5,000 in early 2008. Most residents depend on fishing and traditional small-scale farming. Two rainy seasons occur annually from approximately March to June and October to November, but the periods vary among years. The total annual rainfall in 2007 was 1,179.5 mm, and the average temperature was 23.0°C (maximum: 31.5°C, minimum: 16.0°C). Malaria is the leading cause of morbidity and mortality of children in the region [9].

### Emerged land

The area of the narrow strip of land emerging along the lakeshore was estimated from past and recent shorelines. The past shoreline was reconstructed from remnants of old lake bank that were formed by waves during the period of high lake water in 1998. Landmarks photographed in 1998 and 1999 were also used to reconstruct the shoreline. The photographed landmarks included fences, stonewalls, trees, and mosquito breeding sites near the old shoreline. These sites were revisited in early 2008, and their coordinates were recorded using a hand-held global positioning system (GPS) unit and were plotted on the satellite image taken in June 2006 (0.6-m ground resolution Quick Bird image; Digital Globe, Longmont, CO, USA; the water level was approximately 0.9 m below the average in June 2006) using a geographical information system (GIS; Arc View 9.2; Environmental Systems Research Institute, Redlands, CA, USA). With the exception of most of the breeding sites, most of the landmarks were still visible on the satellite image and were used to correct any GPS errors. The recent shoreline was estimated using the satellite image. The distance between the recent and past shorelines along approximately 6.8 km of shoreline was estimated at 68 points at 100-m intervals using GIS.

### Mosquito habitats

Habitat data during the period of high lake water were obtained from three previous studies [8,10,11]. All stagnant water habitats in the study area were surveyed three times during the rainy seasons (February to March 1998; March and May 1999), and once during the dry season (February 1999). At each habitat, 2 to 50 samples (depending on habitat size) were collected to confirm the presence/absence of anopheline larvae. Sampled larvae were immediately preserved in 96% ethanol.

When numerous small habitats were present in an area, it was considered a single site. The characteristics of habitats were noted, and their locations were recorded using a GPS unit. The survey was repeated three times during the rainy season (March and April 2008) and once during the dry season (February 2008) when the lake water was low.

### Species identification

Sampled larvae were identified under light microscopy using the identification key of Gillies and Coetzee [12]. For the surveys during the period of high lake water, the *Anopheles gambiae *complex and the *Anopheles funestus *complex were further identified to species using an rDNA-polymerase chain reaction (PCR) method [13]. Indistinguishable small individuals were also examined using the PCR method.

For the surveys during the low lake water period, anophelines found in unvegetated habitats were assumed to belong to the *A. gambiae *complex, because previous studies have demonstrated that all anophelines in the habitats of this area were either *Anopheles arabiensis *or *A. gambiae *[8,10,11]. However, two anopheline groups, the *A. funestus *complex and the *Anopheles coustani *complex (non-malaria vector), occur in vegetated habitats, and *A. arabiensis *and *A. gambiae *may also occur in small unshaded habitats, such as animal footprints, associated with large vegetated pools. Thus, large individuals (third or fourth instars) from vegetated habitats were identified under light microscopy. Because *A. funestus *is the only species within its complex known to inhabit the study area, individuals identified as belonging to the *A. funestus *complex were assumed to be *A. funestus*. Thus, the PCR method was only applied to microscopically indistinguishable small individuals from vegetated habitats in later surveys.

## Results

**Figure 2:**
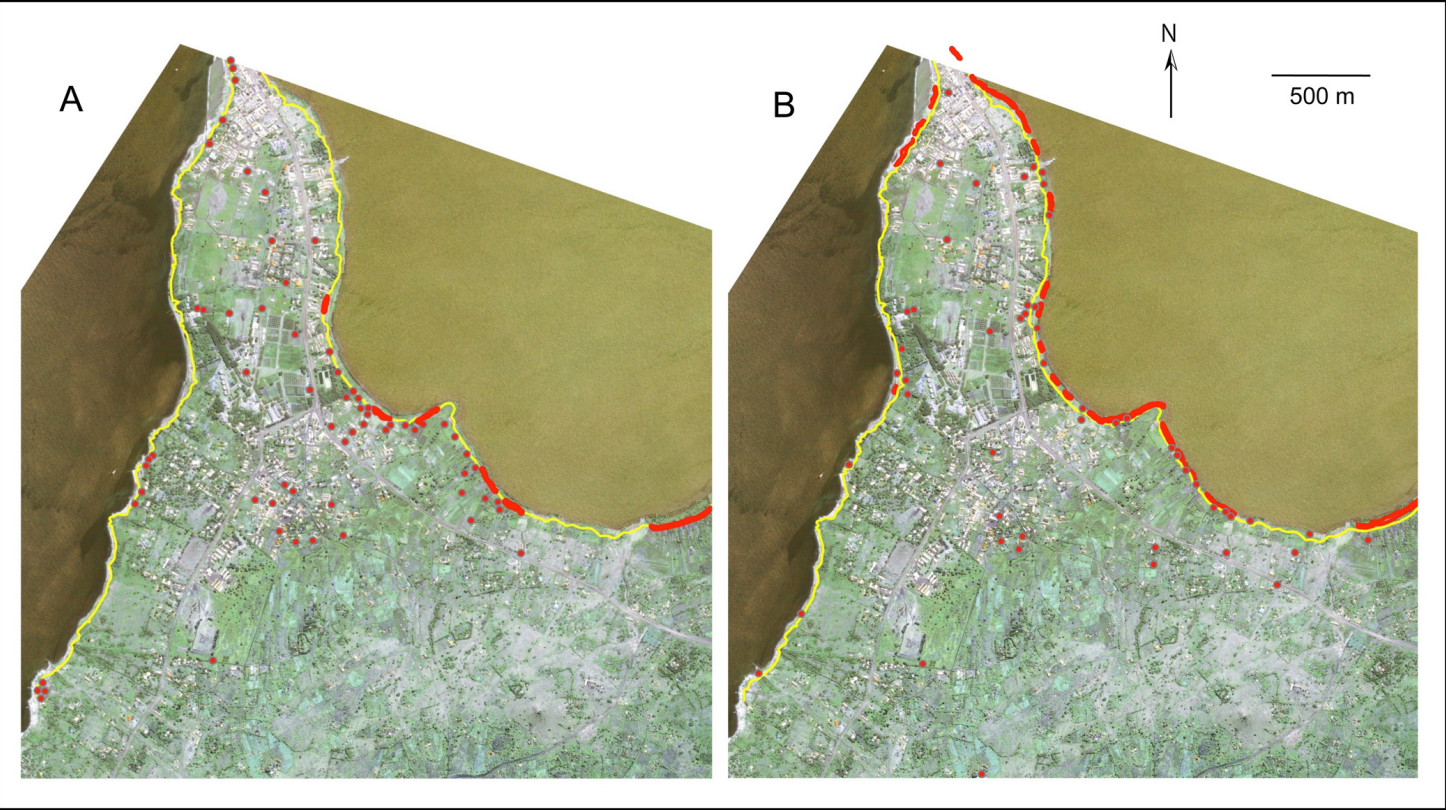
Breeding habitats (red) for malaria vectors found during the high (A) and low (B) lake water periods and the old shoreline (yellow line).

The average distance between the recent and past shorelines was 26.8 m (SE = 2.2 m; range = 5–94 m; n = 68; Figure 2). The estimated area of the newly emerged land was 0.18 km^2 ^(4.7% of the total study area).

Anopheline larvae were found in 69 discrete breeding habitats during the high lake water period (Figure 2). Seven habitats (10.2%) were shaded by emergent aquatic plants, such as sedge (Cladium) or were covered with water hyacinth (*Eichhornia crassipes*) or duckweed (Lemnaceae). All vegetated habitats were found near the lakeshore, and four of these were partially connected to the lake. The other 62 (89.8%) habitats had little vegetation.

In total, 2,840 larvae were examined for species identification, and 2,621 were identified from the surveys during the high lake water period. The species composition included the following: *A. gambiae *(75.0%), *A. arabiensis *(23.4%), *A. funestus *(1.5%), and the *A. coustani *complex (0.1%). Only one vegetated habitat had both *A. funestus *and the *A. coustani *complex but did not have the *A. gambiae *complex. The other 68 (98.6%) habitats had either or both *A. arabiensis *and *A. gambiae*.

During the low lake water period, anopheline larvae were found in 71 discrete habitats (Figure 2), 36 (50.7%) of which were found on the newly emerged land. Twenty-five habitats (35.2%) were vegetated and were all located on the newly emerged land. Of these vegetated habitats, 12 were partially connected to the lake. In addition, 11 unvegetated habitats were found on emerged land. All inland habitats had little vegetation. The proportion of vegetated to unvegetated habitats was significantly higher during the low compared to the high lake water period (chi-squared test: χ^2 ^= 12.09, P < 0.001).

During the low lake water period, 198 larvae from vegetated habitats were examined microscopically. Of these, 29 individuals belonged to the *A. funestus *complex, 12 to the *A. gambiae *complex, 30 to the *A. coustani *complex, and 127 were unidentified. The results of the PCR method applied to the unidentified individuals revealed that 15 individuals were *A. funestus*, 20 were *A. gambiae*, 26 were *A. arabiensis*, and the remaining 66 individuals were unidentifiable. Because the *A. coustani *complex was not analysed using PCR identification, several unidentified individuals may belong to this complex.

*Anopheles funestus *and the *A. coustani *complex were found in 18 and 16 vegetated habitats during the low water period, respectively, and more of their habitats were available during the low water period than the high water period. All habitats of these species were located on the newly emerged land. The proportion of *A. funestus *habitats relative to the total number of habitats was significantly higher during the low (25.4%) compared to the high water period (1.5%; chi-squared test: χ^2 ^= 20.34, P < 0.001). Similarly, the proportion of habitats of the *A. coustani *complex was significantly higher during the low (22.5%) than the high water period (1.5%; chi-squared test: χ^2 ^= 17.31, P < 0.001).

The *A. gambiae *complex was also found in 14 vegetated habitats. In total, 60 of 71 habitats contained the *A. gambiae *complex, which was found in more habitats during the high lake water period. Twenty-five of these habitats were located on the newly emerged land. The proportion of habitats positive for the *A. gambiae *complex was significantly higher during the high (98.5%) than the low water period (84.5%; chi-squared test: χ^2 ^= 10.22, P = 0.001).

## Discussion

**Figure 3:**
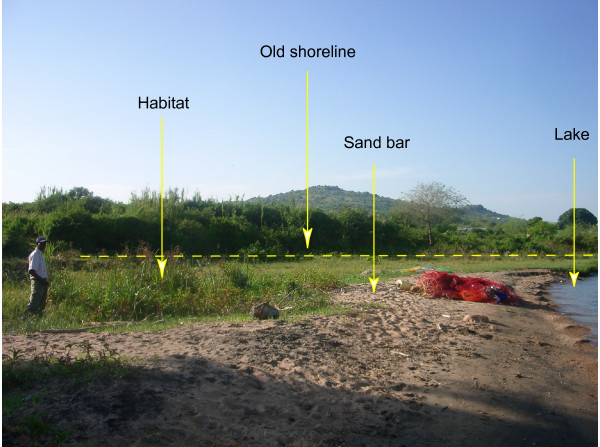
Typical breeding habitat for *Anopheles funestus*, the old shoreline (dotted line), and a sandbar isolating the habitat from the lake.

As the water in Lake Victoria retreated during the last 10 years, a narrow strip of land emerged along the lakeshore. This land was wide and stable enough to sustain several breeding habitats for *A. funestus *and the *A. gambiae *complex. In particular, all identified habitats for *A. funestus *were found on the newly emerged land. Water accumulates in depressions and appears to be supplied through rainfall, seepage from the ground, and occasionally high waves. Some habitats were isolated from the lake by sandbars created by wave action (Figure 3).

Although the total number of habitats during the low lake water period was nearly equal to that during the high water period, the number of habitats for the *A. gambiae *complex was lower during the low lake water period. Because habitat availability for the *A. gambiae *complex largely depends on rainfall [14], it is difficult to determine whether the lake water level affects the availability of habitats for this species. Because the *A. gambiae *complex breeds in small temporal habitats [15], many suitable habitats quickly appear after several heavy rains but disappear during the dry period [14]. In fact, the unusually high amount of rainfall in 1997 and early 1998 created several habitats for the *A. gambiae *complex, whereas only one habitat for *A. funestus *existed during this same period. Habitats for *A. funestus *are stable for longer periods [15,16], and rainfall alone cannot explain the increase in *A. funestus *habitats.

The emerged land was initially bare and lacked dense vegetation. When the lake water level stabilized, short grasses gradually covered the area, and emergent aquatic plants later began to invade the aquatic habitats. Local farmers have been reluctant to farm the land, because it may again become submerged in the future. The lack of human activity was crucial to the appearance of *A. funestus *habitats. When the water level was high in 1998 and 1999, it rose close to houses and farms, such that little space was left to accumulate enough water for *A. funestus *to breed, although small puddles were sufficient for breeding of the *A. gambiae *complex. However, if the lake water level remains low, farmers will begin to convert the vegetated aquatic habitats to farmland. Consequently, this activity may convert the habitats for *A. funestus *to habitats for the *A. gambiae *complex by creating small sun-lit open puddles and ditches that lack tall aquatic vegetation [17,18].

## Conclusion

These findings clearly indicate that the recent reduction in the water of Lake Victoria has increased the number of habitats for *A. funestus*. Although the results should not be simply extrapolated to other areas, the water reduction is likely to substantially affect the populations of malaria vectors in the Lake Victoria basin, particularly because Lake Victoria is the second largest freshwater body in the world and has numerous islands and a long shoreline in an area to which malaria is endemic. The availability of habitats is usually more limited for *A. funestus *compared to the *A. gambiae *complex, but the recent water drop may increase the role of *A. funestus *in malaria transmission in the basin.

## Authors' contributions

NM initiated the study and drafted the manuscript. GS and GD led the field survey. KF and SK analysed the data. All authors have read and approved the final manuscript.
